# Orbital myositis: a report of 3 pediatric cases

**DOI:** 10.1186/1546-0096-10-S1-A92

**Published:** 2012-07-13

**Authors:** Gaëlle Chédeville, Rosie Scuccimarri

**Affiliations:** 1 The Montreal Children's Hospital, Montreal, QC, Canada

## Purpose

Orbital myositis is a localized form of idiopathic orbital inflammation, which is a benign inflammatory condition involving the structures of the orbit. Although the majority are idiopathic, association with systemic disease has been reported. This diagnosis is particularly rare in children. Herein we report 3 pediatric patients with orbital myositis.

## Results

### Case 1

An 8 yo boy was referred to our clinic in March 2008 for 2 episodes of orbital myositis. The first episode occurred in November 2007 and involved the left eye. Investigations with a CT scan and an MRI revealed changes in the left lateral rectus muscle, felt to be consistent with a myositis. This spontaneously resolved over a few days. In January 2008, the child’s right eyelid became swollen and he had an associated diplopia. The MRI revealed a normal left rectus muscle but inflammation was observed in the right rectus muscle. Again, without treatment, the symptoms resolved. It was at that time that the patient was referred to Rheumatology. On review of systems, he was noted to have longstanding epigastric pain associated with meals. His physical examination was unremarkable. Blood tests revealed iron deficiency, microcytic anemia and an elevated ESR. He was referred to Gastroenterology. He underwent an endoscopy which confirmed the diagnosis of Crohn’s disease. There has been no recurrence of the orbital myositis since the Crohn’s disease has been in remission.

### Case 2

A 15 yo girl presented with acute onset of right eye swelling in May 2010. Six months prior, she had a similar episode that lasted for one week and resolved on its own. Imaging studies revealed changes in the right medial rectus muscle consistent with myositis. A review of systems was completely unremarkable. The physical examination revealed only a right upper eyelid swelling. A full work-up was done which was completely normal. She was started on oral steroids and had a very good response within days. She remains well off prednisone and has not developed any systemic symptoms.

### Case 3

A 14 yo boy presented with left eye swelling in June 2010. The diagnosis of orbital myositis was confirmed on CT scan, which showed involvement of all the extra-ocular muscles, mainly the medial rectus. The review of systems was significant for recurrent mouth ulcers and poor growth. A work-up was performed which was normal except for a mild anemia and an elevated ESR. Symptoms resolved on prednisone. Six months later, he presented with a new episode of left orbital myositis. On examination, he had marked oral ulcers with significant lip swelling. He underwent an endoscopy which did not demonstrate any signs of colonic inflammation. Due to a high suspicion for Crohn’s disease, the patient is now awaiting a capsule endoscopy. Once again, he has had an excellent response to prednisone.

## Conclusion

Orbital myositis is a rare condition in children. These cases are usually referred to Rheumatology to investigate for a possible underlying condition and for initiation of immunosuppressive therapy. Although most cases are idiopathic, a complete work-up is warranted in these patients.

## Disclosure

Gaëlle Chédeville: None; Rosie Scuccimarri: None.

